# Exogenous estradiol improves shell strength in laying hens at the end of the laying period

**DOI:** 10.1186/1751-0147-56-34

**Published:** 2014-05-27

**Authors:** Anna Wistedt, Yvonne Ridderstråle, Helena Wall, Lena Holm

**Affiliations:** 1 Department of Anatomy, Physiology and Biochemistry, SLU, Swedish University of Agricultural Sciences, Box 7011, Uppsala SE-750 07, Sweden; 2 Department of Animal Nutrition and Management, SLU, Swedish University of Agricultural Sciences (Kungsängen Research Center), Uppsala SE-753 23, Sweden

**Keywords:** Exogenous estradiol, Eggshell formation, Carbonic anhydrase, Estrogen receptors, Bone strength, Eggshell quality, Domestic hen

## Abstract

**Background:**

Cracked shells, due to age related reduction of shell quality, are a costly problem for the industry. Parallel to reduced shell quality the skeleton becomes brittle resulting in bone fractures. Calcium, a main prerequisite for both eggshell and bone, is regulated by estrogen in a complex manner. The effects of estrogen, given in a low continuous dose, were studied regarding factors involved in age related changes in shell quality and bone strength of laying hens. A pellet containing 0.385 mg estradiol 3-benzoate (21-day-release) or placebo was inserted subcutaneously in 20 birds each of Lohmann Selected Leghorn (LSL) and Lohmann Brown (LB) at 70 weeks of age. Eggs were collected before and during the experiment for shell quality measurements. Blood samples for analysis of total calcium were taken three days after the insertion and at sacrifice (72 weeks). Right femur was used for bone strength measurements and tissue samples from duodenum and shell gland were processed for morphology, immunohistochemical localization of estrogen receptors (ERα, ERβ), plasma membrane calcium ATPase (PMCA) and histochemical localization of carbonic anhydrase (CA).

**Results:**

Estrogen treatment increased shell thickness of both hybrids. In addition, shell weight and shell deformation improved in eggs from the brown hybrids. The more pronounced effect on eggs from the brown hybrid may be due to a change in sensitivity to estrogen, especially in surface epithelial cells of the shell gland, shown as an altered ratio between ERα and ERβ. A regulatory effect of estrogen on CA activity, but not PMCA, was seen in both duodenum and shell gland, and a possible connection to shell quality is discussed. Bone strength was unaffected by treatment, but femur was stronger in LSL birds suggesting that the hybrids differ in calcium allocation between shell and bone at the end of the laying period. Plasma calcium concentrations and egg production were unaffected.

**Conclusions:**

A low continuous dose of estrogen improves shell strength but not bone strength in laying hens at the end of the laying period.

## Background

Age related reduction in shell quality is a costly problem for the table egg industry. Eggshell consists mainly of calcium carbonate (CaCO_3_) and the ionic precursors are supplied by the blood through trans-epithelial transport. Blood flow through the shell gland increases dramatically during shell formation [1] and previous experiments in our laboratory show a connection between capillary density and shell quality [2].

Carbonic anhydrases (CA) have been implicated in shell formation for nearly 70 years [3], when Benesch and co-workers demonstrated that inhibition of CA in laying hens results in soft-shelled or shell-less eggs. The enzyme catalyzes the hydration of metabolic CO_2_ to HCO_3_^-^, the precursor of eggshell carbonate. Since carbonate transport is, to some extent, coupled to calcium transport in the shell gland mucosa, CA is involved in providing both of the major constituents for shell formation [4]. CA is regulated by estrogen in the endometrium of mice and guinea-pigs [5,6]. In laying hens, the phytoestrogen daidzein increases the number of CA positive capillaries in the shell gland of Lohmann Brown hybrids [7] and embryonic exposure to estrogenic substances also affects shell gland capillary CA [8]. Thus, evidence exists suggesting that regulation of CA by estrogen may take place also in the laying hen shell gland.

Eggshell calcium is derived from duodenal absorption, which increases 6-fold during shell formation [9]. However, skeletal stores are needed as a secondary source resulting in a constant remodeling of the bone structure in laying hens, which reduces bone strength and increases the incidence of fractures. Many laying hens, especially those housed in cages, suffer from these fractures making it a welfare problem that requires further investigation [10].

It is well known that estrogen is implicated in shell formation indirectly by acting on organs involved in calcium metabolism and an injection of estradiol increases the circulating levels of calcium in plasma [11]. Estrogen also up-regulates one of the main pathways for calcium transport at the cellular level, the ATP-dependent plasma membrane calcium pump (PMCA) [12,13]. If the hormone has a more direct effect on the shell gland is still unclear, however, exogenous estrogen at a dose of 10 μg/kg body weight (bw) increases eggshell thickness in Tegel pullets [14] and a supplement of daidzein improves shell quality of eggs from ISA layers [15]. Furthermore, we have shown that both estrogen receptors (ERα and ERβ) are present in the functional shell gland of laying hens [7] and preliminary data from our group show that a shift in the balance between these receptors coincides with reduced shell quality.

The aim of the present experiment was therefore to study the effect of a low dose of estradiol, provided by a subcutaneous pellet with a 21 day release, on shell and bone quality in laying hens at the end of a laying period and on a number of key factors involved in duodenal calcium absorption and shell formation in the shell gland.

## Materials and methods

### Animals, housing and management routines

Laying hens of two different hybrids, 20 Lohmann Selected Leghorn (LSL) and 20 Lohmann Brown (LB) (Gimranäs AB, Sweden) arrived at the poultry house at 15 weeks of age. The birds were held in the university poultry research facility under conditions similar to commercial egg production. The birds were housed in groups of five hens in furnished 8-hen cages as described by Wall and Tauson [16]. The light was gradually increased from 9 h/24 h until 14 h/24 h was obtained at 23 weeks of age. The hens were fed according to a phase feeding diet for commercial hens, distributed by a Swedish feed manufacturer (Lantmännen) and had free access to water. At 70 weeks of age, a pellet containing 0.385 mg estradiol 3-benzoate or a placebo pellet (Innovative Research of America, Sarasota, FL, USA) was implanted in 10 birds of each hybrid subcutaneously in the neck. The pellets had a 21 day release of estradiol 3-benzoate resulting in a daily dose of around 9–11 μg/kg bw depending on hybrid. The cages fulfilled the Swedish Animal Welfare Directives and the study was approved by the Uppsala Local Ethics Committee.

### Eggshell measurements

Eggs were collected seven hours after lights-on each morning and number of eggs and egg weights were recorded five days before the insertion and during the entire experimental period. Eggs collected before the experimental period were used to establish base-line values. Laying % was calculated as number of eggs laid/day and hen x 100. Each egg was marked on three points approximately 120° apart along the equator of the egg with a pencil. Shell deformation was calculated from the average value of measurements on each marking point, after a load of 1,000 g was applied on the egg (The Canadian Egg Shell Tester, Otal Precision Company Ltd, Ottawa Ontario, Canada K1G3N3). The shell breaking strength was recorded for each egg using the same instrument. The shell pieces were then rinsed clean of albumen and yolk with distilled water and dried over night at 120°C. Shell weight including shell membranes was recorded. Shell thickness was measured using a digital micrometer (Mitutoyo Absolute, No. 7360; Mitutoyo Corp., Stockholm, Sweden) at the three marked points along the equator. Shell membranes were removed by boiling the shells in 2.5% (w/v) NaOH for 8 min, rinsing in distilled water and drying overnight at 120°C. Shell thickness without membranes was then measured according to the same procedure as above.

### Tissue preparation

The hens were killed at 72 weeks of age by an intravenous injection of pentobarbital sodium (100 mg/ml, Apoteket AB, Umeå, Sweden) in the wing vein and bw was recorded for each bird. Left oviduct and small intestine were rapidly removed. The oviduct was dissected free from the mesoviductus and straightened, the length was measured from the vaginal orifice to the fimbriated infundibulum, and the location of egg was recorded. The shell gland was then cut open lengthwise and pieces cut from the middle part were removed for fixation. A 2 cm piece of small intestine immediately distal to the duodenal loop was removed and cut open. All tissue pieces were divided in two and pinned to small rectangles of cork to minimize tissue distortion. One piece of each tissue was fixed in 2.5% glutaraldehyde in 0.067 M phosphate buffer (pH 7.2) and the other piece in 4% paraformaldehyde in 0.067 M phosphate buffer (pH 7.2) for 24 h at 4°C. After rinsing in phosphate buffer the tissue was trimmed into 2 mm thick transverse slices. Following dehydration in increasing concentrations of ethanol, samples were embedded both in a water-soluble resin (Leica Historesin, Heidelberg, Germany) for CA histochemistry and paraffin for immunohistochemistry.

### Breaking strength of the femur

The right leg was frozen at sacrifice and stored until analysis. Before measurement, the leg was thawed to room temperature. Femur was free dissected from skin, ligaments and muscles and tested to the breaking point on a three-point bending electromechanical material testing machine (Avalon Technologies, Rochester, MN, USA). The loading speed was 1 mm/sec and the span length used was 30 mm. The load was applied to the mid-diaphyseal part of the bone. Digital data were collected 50 times per second until failure using software provided with the testing machine (Testware II).

### Total calcium in plasma

Blood samples were collected three days after implant insertion and just prior to sacrifice and were held on ice for approximately two hours before centrifugation at 3000 rpm at 4°C for 10 min. Plasma was collected and stored at -70°C until analysis of total calcium (mmol/l) was performed (Calcium Architect cSystems, Aeroset System, Abbott Laboratories, Solna, Sweden).

### CA histochemistry

Histochemical localization of CA activity was performed according to Ridderstråle’s histochemical method [17]. Sections (2 μm) of resin embedded tissues were cut on a microtome (Leica RM 2165, Leica Instruments, Germany) using glass knives. The sections were incubated for 6 min floating on the incubation medium containing 3.5 mM CoSO_4_, 53 mM H_2_SO_4_, 11.7 mM KH_2_PO_4_ and 157 mM NaHCO_3_. After incubation the sections were rinsed in 0.67 mM phosphate buffer (pH 5.9), transferred to 0.5% (v/v) (NH_4_)_2_S, and finally rinsed with two successive baths of distilled water. The incubation procedure results in a black precipitate of cobalt sulphide at sites with CA activity. Before mounting, some of the sections were counter-stained with azure blue. Neighboring sections were stained with hematoxylin-eosin for conventional histology. The specificity of the reaction was checked using the CA inhibitor acetazolamide. Sections were first preincubated on a 10 μM solution of acetazolamide for 30 min and then incubated as above but with an incubation medium containing 10 μM inhibitor.

### Immunohistochemistry

The paraffin embedded tissues were cut into 4 μm thick sections and mounted on Superfrost Plus Gold slides, (Menzel-Glaser, Braunschweig, Germany), deparaffinized in xylene and rehydrated in graded alcohol. The sections were rinsed in PBS buffer after each step in the following procedure. Antigen retrieval was performed by pressure heating for 20 minutes in a pressure-boiler (21100 Retriever, Histolab Products AB, Gothenburg, Sweden) using 0.01 M sodium citric buffer (pH 6.0). Endogenous peroxidase activity was blocked with 3% hydrogen peroxide. Endogenous avidin and biotin activity were blocked (Vector Laboratories, Inc., Burlingame, CA, USA) in the assay for ERβ. All sections were treated with normal serum provided by the kit for the secondary antibody used in the respective assay (see below).

The sections for primary antibodies were incubated in the dark at room temperature for two hours for PMCA, and at +4°C overnight for ERα and ERβ. ERα was detected using a rabbit anti-ERα (clone 60C, Millipore, USA) diluted 1:50. ERβ was detected using a mouse monoclonal antibody (MCA 1974ST, Serotec, Düsseldorf, Germany) diluted 1:20 and PMCA was detected using a mouse monoclonal antibody (5 F10 ab2825, Abcam 330, Cambridge Science Park, Cambridge, CB4 0FL, UK) diluted 1:1000.

The secondary antibody used in the assays for ERα and PMCA was a peroxidase labeled anti-rabbit or anti-mouse IgG (Cat. No. MP-7401 and MP-7402, ImmPRESS reagent kit, Vector Laboratories, Burlingame, CA, USA). The secondary antibody used in the assay for ERβ was a biotinylated secondary horse anti-mouse IgG and these sections were incubated with a peroxidase-avidin-biotin complex (Vectastain ABC kit; PK6102 Elite, Vector Laboratories, Inc., Burlingame, CA USA). All sections were treated with the chromogen 3.3′-diaminobenzidine tetrahydrochloride (DAB-safe, Saveen Biotech, Malmö, Sweden) to which H_2_O_2_ was added to visualize the bound enzyme activity as a brown color.

Negative controls were run by omitting the primary antibodies and by replacing the primary antibody with non-immune serum from mouse (sc-2025) in the ERβ and PMCA analyses, and from rabbit (sc-2027) in the ERα analyses (Santa Cruz Biotechnology, Inc., Santa Cruz, CA. 95060 USA). Sections from rooster epididymis were used as positive controls for ERα and from pig uterus for ERβ. All sections were mounted with Pertex (Histolab products AB, Gothenburg, Sweden).

### Image analysis and morphometric evaluation

All slides were coded before examination and only areas free from artifacts were chosen. Digital images of CA-stained sections from shell gland were taken with a Nikon Microphot-FXA microscope using the 10x objective lens.

For evaluation of CA activity in the shell gland, one image from each top of five consecutive mucosal folds, containing mucosal surface epithelium and sub-epithelial tubular glands were analyzed, i.e. five images/section and hen. Only mucosal folds attached to the underlying submucosal layer were chosen for analysis. The total number of capillaries/mm^2^, number of CA positive capillaries/mm^2^ were recorded using an image analysis software (Elcipse Net, version 1.20, Developed by Laboratory Imaging, Prague, Czech Republic). Capillaries were counted only when their entire circumference was located within the picture frame. A capillary was considered CA positive when more than half of its circumference showed CA activity (black staining). The sections containing CA positive tubular gland cell membrane was noted.

For evaluation of CA activity in the duodenum, membrane bound and cytosolic activity was noted and the staining intensity was scored on a scale from 1–3. Score 1: weak staining or absent, score 2: intermediate and score 3: strong staining.

For the immunohistochemical evaluation of ERα and ERβ in the shell gland, the localization was described and the staining of each structure on one slide/bird was scored for intensity on a scale from 0–3, where zero corresponds to no staining and three representing strong staining.

No differences in localization or intensity were observed regarding the immunohistochemical staining of ERα and ERβ in the duodenum and PMCA in the shell gland and duodenum and these sections were therefore not subjected to further measurements.

### Statistical analysis

In the Morphometric measurements, staining intensity was graded from 0 (no staining) to 3 (strong). Each variable in the different tissue was analyzed statistically by the general linear model (Proc GLM) using SAS® (SAS Institute Inc., Cary, NC, USA, version 9.2). All other data were analyzed statistically by (Proc Mixed) using SAS® and Bonferroni corrections for multiple comparisons were used.

The statistical models included the fixed effect of hybrid (n = 2), treatment (n = 2) and interaction between fixed effects. Egg weight was included as a covariate in the analysis of shell weight and shell weight was included as a covariate in the analysis of shell thickness. In the analyses of production and eggshell quality traits, each group, i.e., hens housed in the same cage, was treated as one experimental unit.

In the analysis of body weight, organ measurements, morphological data, total calcium and bone strength each hen was treated as an experimental unit, i.e. ten replicates per hybrid and treatment. Body weight was included as a covariate in the analyses of bone strength. Results are given as mean ± SE unless otherwise stated. A difference was considered significant at *P* < 0.05.

## Results

### Body weight and organ measurements

The body weight as mean ± SE (n = 20) was 2052 ± 53.8 g (LB) and 1707 ± 32.0 g (LSL), the LB hens were heavier compared to LSL hens (*P* < 0.0001). Body weight was not affected by treatment or interaction between treatment and hybrid. All hens had a well developed left oviduct with an average length of 58 cm and fully developed follicles in the ovary. The oviduct length did not differ between treatment or hybrids and no interaction was found between treatment and hybrid. The ratio between left oviduct and body weight was higher in the LSL hens compared to LB hens (p = 0.0003). The ratio was not affected by the treatment or interaction between treatment and hybrid.

### Egg production and shell quality

Laying % during the experimental period as mean ± SE (n = 40) was 86 ± 1.4% (LB and LSL) was not affected by the estradiol implant or differed between hybrids. There was no interaction between hybrid and treatment. The estradiol treatment clearly improved eggshell quality in eggs from the LB hybrids (Table 1). In these eggs shell deformation decreased, shell weight and shell thickness with and without shell membranes increased. Eggs from the LSL hens had improved shell thickness when shell membranes were removed and showed a tendency for thicker shell with membranes, while other quality parameters were unaffected. The brown LB eggs were heavier compared to the white LSL eggs and breaking strength was stronger in LB eggshells compared to LSL eggshells. The parameters measured in eggs collected the week prior to insertion of the pellet did not differ from eggs from the placebo groups i.e. the insertion of the pellet had no effect in itself on production and eggshell quality.

**Table 1:** Eggshell quality measurements from hens after estradiol 3-benzoate or placebo treatment

	**Lohmann Selected Leghorn (LSL)**			**Lohmann Brown (LB)**		
	**Placebo**	**Estradiol**	***P *** **value**	**Placebo**	**Estradiol**	***P *** **value**
	**(n = 2)**	**(n = 2)**		**(n = 2)**	**(n = 2)**	
Egg weight (g)	65.9 ± 0.35	65.6 ± 0.46	n.s	67.0 ± 0.50	66.3 ± 0.37	n.s
Shell Deformation (μm)	74 ± 0.9	73 ± 0.9	n.s	74 ± 1.2	69 ± 0.7	0.0002
Shell breaking strength (g)	4077 ± 71	4026 ± 58	n.s	4263 ± 79	4388 ± 71	n.s
Shell weight (g)*	5.94 ± 0.04	5.96 ± 0.04	n.s	6.09 ± 0.04	6.38 ± 0.04	<0.0001
Shell thickness incl membrane (mm)*	0.386 ± 0.002	0.390 ± 0.002	0.0749	0.400 ± 0.002	0.409 ± 0.002	0.0014
Shell thickness excl membrane (mm)*	0.357 ± 0.002	0.362 ± 0.002	0.0373	0.372 ± 0.002	0.378 ± 0.002	0.0415

Eggshell quality of eggs from Lohmann Selected Leghorn (LSL) and Lohmann Brown (LB) hens treated with estradiol 3-benzoate or a placebo pellet at the end of the laying period. Values are expressed as mean ± SE.

*Egg weight was included as a covariate in the analysis of shell weight and shell weight was included as a covariate in the analysis of shell thickness, these values are expressed as LSmean ± SE.

### Bone breaking strength

**Figure 1:**
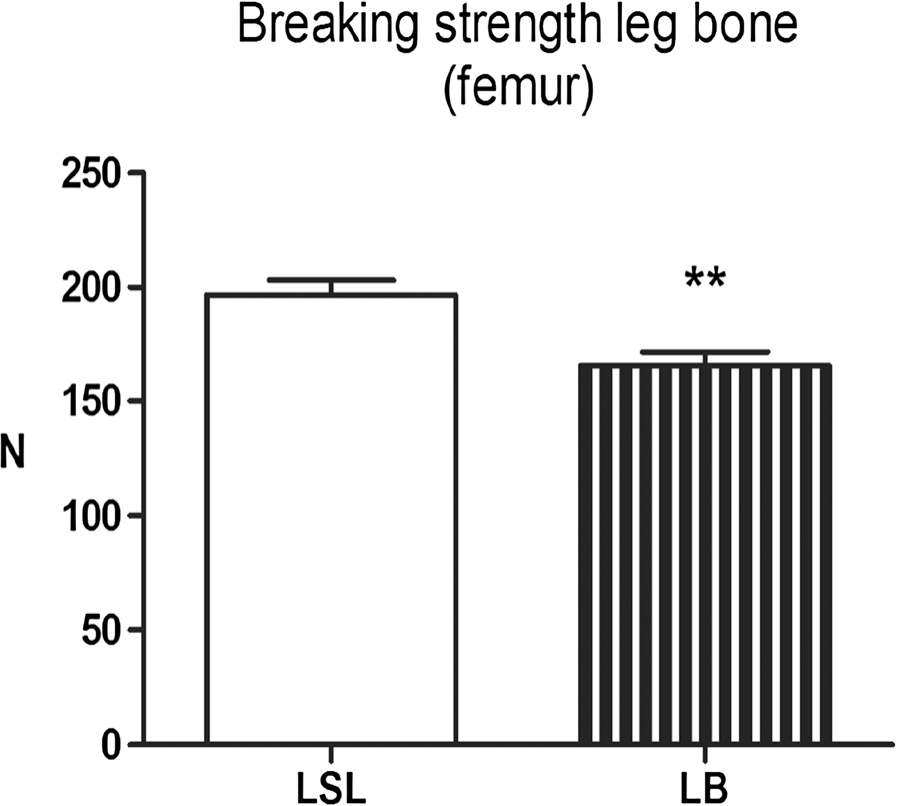
**Breaking strength of leg bone (femur) tested by three-point bending, load at failure (Newton), from Lohmann Selected Leghorn (LSL) and Lohmann Brown (LB) hens (n = 20) at the end of a laying period.** Results are expressed as LSmean ± SE. **(*P* < 0.01).

Bone strength of the femur was not affected by the estradiol implant. The LSL hens had a stronger femur compared to LB hens (Figure 1).

### Total calcium in plasma

Total calcium in plasma as mean ± S.E. (n = 20) was 7.28 ± 0.219 mmol/l (3 days after insertion of estradiol implant) and 7.35 ± 0.279 mmol/l (3 days after insertion of placebo). At sacrifice the calcium concentration was 7.44 ± 0.248 mmol/l in the birds treated with estradiol and 7.42 ± 0.255 mmol/l in birds with a placebo implant. There was no difference in total calcium between the first blood sample and the second blood sample or after treatment with the estradiol. There was no difference between the hybrids or interaction between hybrid and treatment.

### Morphometric measurements

The total number of shell gland capillaries/mm^2^ at the top of the mucosal fold as mean (n = 20) was 363 ± 9.39 (LSL) and 296 ± 7.9 (LB), the total number of capillaries was higher in the LSL hens compared to LB hens (*P* = 0.0027). There was no difference between treatments with estradiol or placebo pellet or interaction between hybrid and treatment.

### Carbonic anhydrase

The histological method used to analyze CA results in a black precipitate at sites of enzyme activity. Sections incubated with the inhibitor contained no significant staining.

#### *Shell gland*

**Figure 2:**
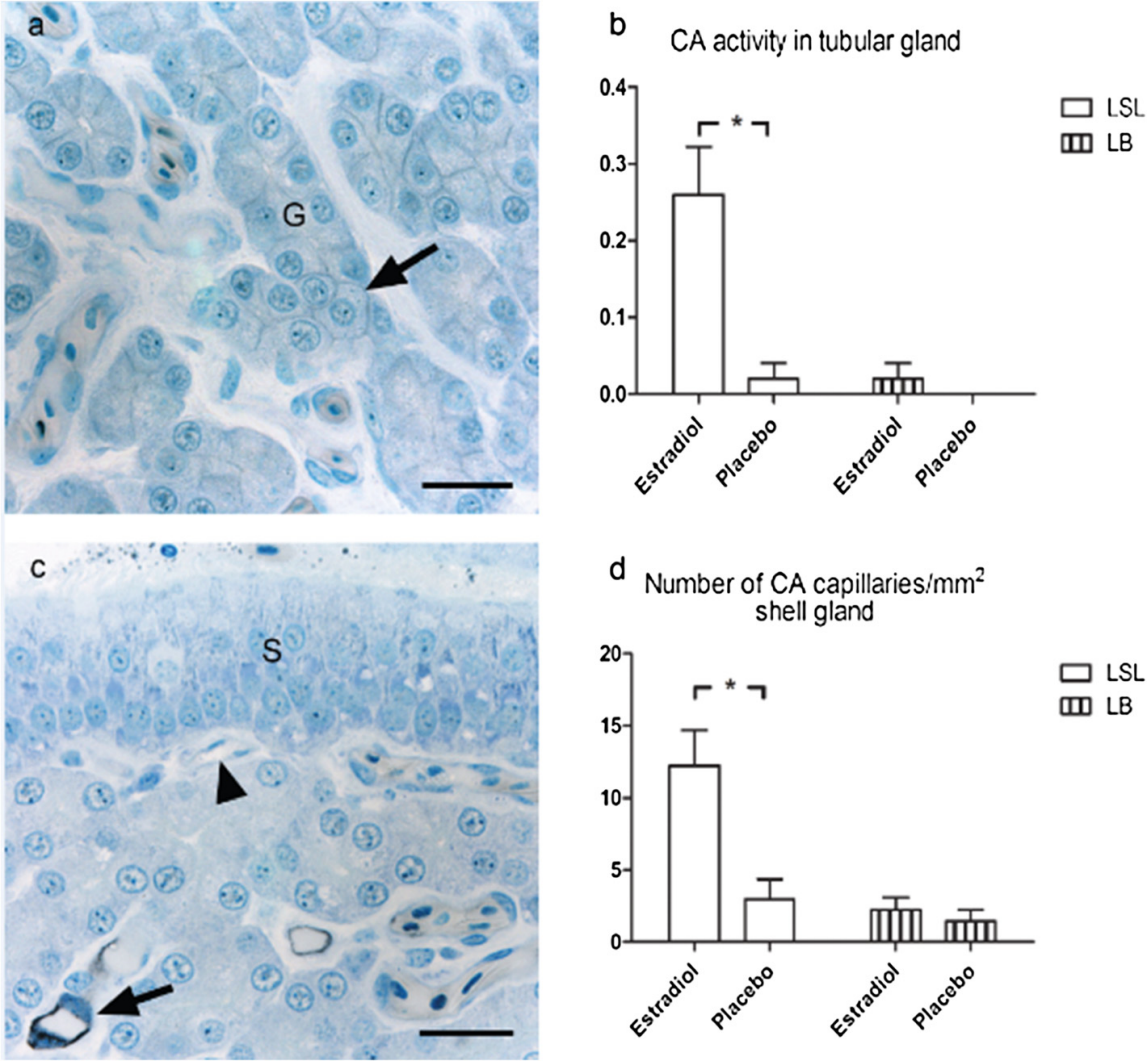
**Localization of carbonic anhydrase (CA) activity in shell gland shown as black staining.** Laying hens Lohmann Selected Leghorn (LSL) and Lohmann Brown (LB) treated with estradiol 3-benzoate or a placebo pellet at the end of the laying period. **(a)** Shell gland mucosal fold of 72-week-old LSL hen treated with estradiol pellet. Weak membrane-bound staining of tubular gland cells (G) (arrow) Bar = 20 μm. **(b)** Amount of CA staining in tubular gland cells of LSL and LB hens (n = 10). Results are expressed as mean ± SE. *(*P* < 0.05). **(c)** Shell gland mucosal fold of 72-week-old LSL hen treated with estradiol. No detectable staining in surface epithelium (S), capillaries with intense membrane-bound staining of endothelial cells (arrow) and capillaries with no staining (arrowhead). Bar = 20 μm. **(d)** Number of CA positive capillaries/mm^2^ in shell gland of LSL and LB hens (n = 10). Results are expressed as mean ± SE. *(*P* < 0.05).

The surface epithelium was unstained in all hens regardless of treatment or hybrid. A minor part of the tubular glands showed weak membrane bound staining for CA activity (Figure 2a). The treatment with estradiol increased the distribution of membrane bound tubular gland CA in the LSL hens compared to the hens treated with placebo, while the LB hens were unaffected by estradiol (Figure 2b).The capillary endothelium showed intense membrane bound staining in some but not all capillaries (Figure 2c) and was calculated in the top of the shell gland mucosal fold. The number of CA positive capillaries increased with estradiol treatment in the LSL hens compared to hens treated with placebo, while the LB hens were not affected by estradiol (Figure 2d).

#### *Duodenum*

Surface epithelium of villi showed intense staining for CA activity in the lateral cell membranes and brush border. Cytosolic staining was moderate. The staining of surface epithelial cells gradually decreased towards the top of the villus. Capillaries with intense membrane bound staining were found in villi, crypt region and muscularis. In the crypt of Lieberkühn lateral cell membranes showed weak, moderate or intense staining, the cytosol showed weak to moderate staining. In the cells of circular muscularis, membrane-bound staining was weak to moderate.

**Figure 3:**
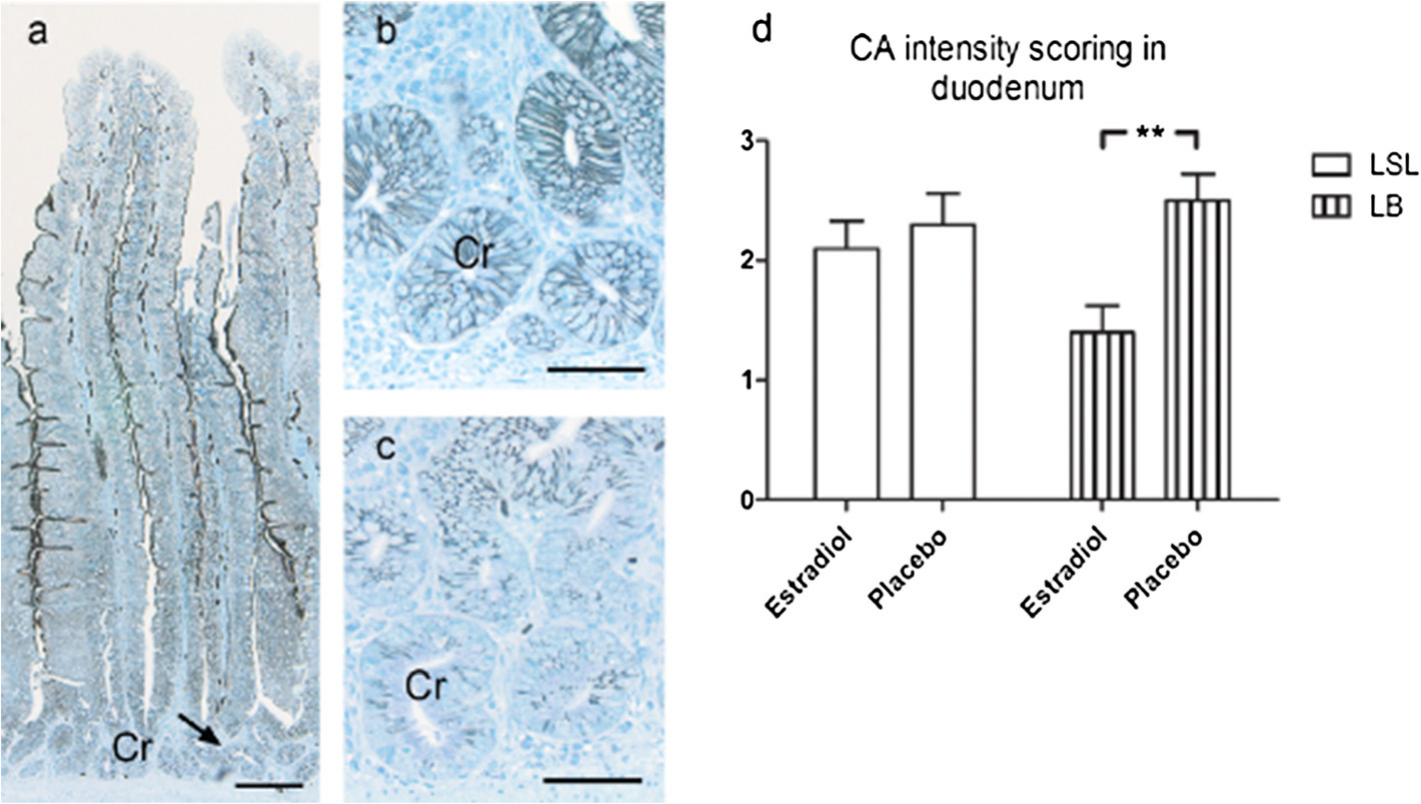
**Localization of carbonic anhydrase (CA) activity in duodenum shown as black staining.** Laying hens Lohmann Selected Leghorn (LSL) and Lohmann Brown (LB) treated with estradiol 3-benzoate or a placebo pellet at the end of the laying period. **(a)** Duodenum of 72-week-old LB hen treated with placebo pellet. Strong membrane-bound staining for CA activity in the lateral cell membranes and brush border of surface epithelium. Capillaries with intense membrane-bound staining in lamina propria, crypt region and muscularis. In the crypts of Lieberkühn membrane-bound staining is moderate to strong. Bar = 160 μm. **(b)** Higher magnification of the same treatment with moderate to strong membrane-bound staining in the crypts of Lieberkühn. Bar = 50 μm. **(c)** Duodenum of LB hen treated with estradiol pellet, with weak or almost absent membrane-bound staining in crypts of Lieberkühn. Bar = 50 μm. **(d)** Intensity scoring of carbonic anhydrase (CA) activity in duodenum. Score 1: weak or absent staining, score 2: intermediate staining, score 3: strong staining. Results are expressed as mean ± SE. **(*P* < 0.01).

The intensity of CA activity in the brush border was stronger in the LB hens compared to LSL hens (*P* = 0.0089). The intensity of CA activity in the lateral cell membrane in the crypts of Lieberkühn decreased with estradiol treatment in the LB hens compared to hens treated with placebo, while the LSL hens were not affected by estradiol (Figure 3).

### Estrogen receptor α

Immunohistochemical localization of ERα in the epididymis from rooster was used as positive control tissue and the result were in agreement with results presented by Oliveira *et al.,*[18]. The negative control showed no staining of significance.

#### *Shell gland*

**Figure 4:**
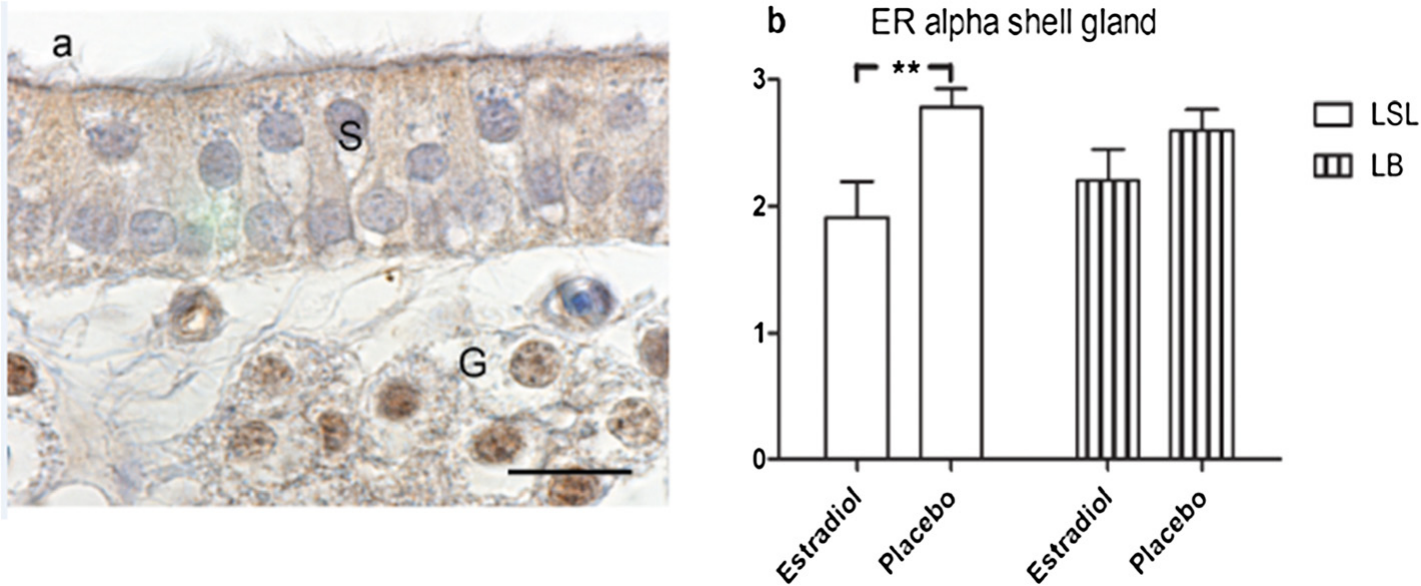
**Estrogen receptor α (ERα) immunolocalization in shell gland after estradiol 3-benzoate or placebo treatment. (a)** Shell gland mucosal fold of Lohmann Brown (LB) hen treated with estradiol pellet, show weak cytosolic granular staining for ERα in surface epithelial cells (S) and unstained nuclei in both ciliated cells and non-ciliated cells. Tubular gland (G) cells show strong nuclear staining for ERα and weak cytosolic staining. Bar = 15 μm. **(b)** Intensity scoring of ERα in shell gland mucosal folds in Lohmann Selected Leghorn (LSL) hen and Lohmann Brown (LB) hens (n = 10). Score 1: weak or absent staining, score 2: intermediate staining, score 3: strong staining. Results are expressed as mean ± SE. **(*P* < 0.01).

In the tubular gland cells strong staining for ERα was found in the nuclei and weak staining in the cytosol. The nuclei of the surface epithelial cells were unstained but a weak to moderate cytosolic granular staining was found in the apical part of both non-ciliated and ciliated cells (Figure 4a).

The endothelium of capillaries was negative but in larger blood vessels the endothelium contained both stained and unstained nuclei. Smooth muscle cells of muscularis and blood vessels had weak to moderate nuclear staining for ERα.In general the staining intensity for ERα in the LSL hens decreased with estradiol treatment compared to hens treated with placebo, while the LB hens were unaffected by estradiol (Figure 4b).

#### *Duodenum*

In duodenum, only weak staining for ERα was found in the nuclei of smooth muscle cells of muscularis and some nuclei of cells in the lamina propria. There were no detectable differences between treatments, hybrids or interaction between treatments and hybrids. No staining for ERα was found in surface epithelial cells of villi or crypts of Lieberkühn.

### Estrogen receptor β

Uterus and cervix from pig was used as positive control tissue and the results were in agreement with results presented by Norrby *et al.,*[19]. The negative controls showed no staining of significance.

#### *Shell gland*

**Figure 5:**
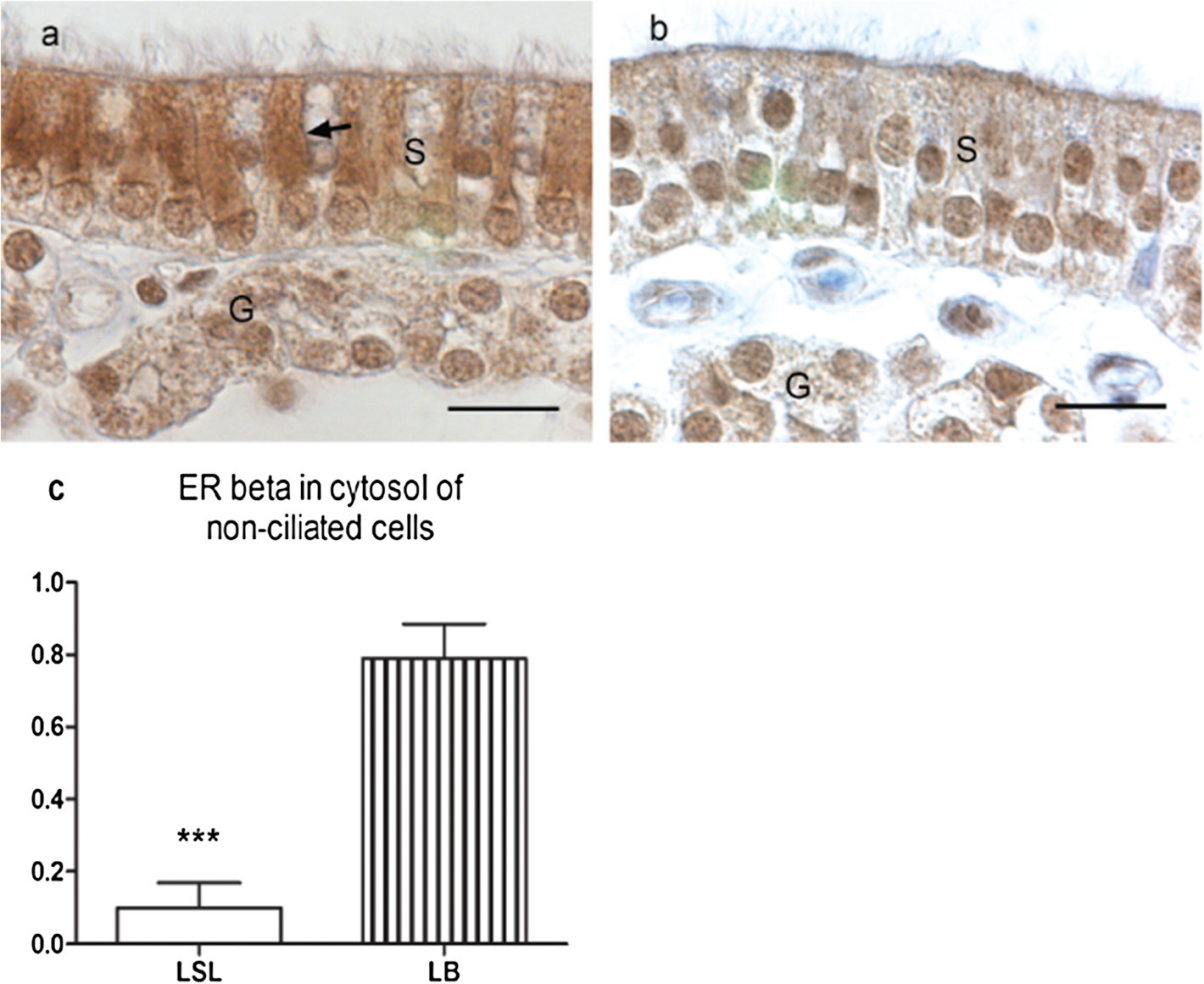
**a-c - Estrogen receptor β (ERβ) immunolocalization in shell gland after estradiol 3-benzoate or placebo treatment.** Strong nuclear staining for ERβ in non-ciliated cells of the surface epithelium (S). Moderate to strong nuclear staining in ciliated cells but also some negative nuclei. Cytosolic staining was found in the surface epithelium and was more pronounced in the non-ciliated cells giving a stripy appearance in the Lohmann Brown (LB) hens compare to Lohmann Selected Leghorn (LSL) hens. Tubular gland (G) cells show moderate to strong nuclear staining for ERβ and moderate cytosolic staining. **(a)** LB hen treated with estradiol, non-ciliated cell with strong cytosolic staining (arrow) **(b)** LSL hen treated with estradiol, non-ciliated and ciliated cells with equal cytosolic staining. **(c)** Amount of cytosolic staining for ERβ in non-ciliated cells in LSL and LB hens (n = 20). Results are expressed as mean ± SE. ***(*P* < 0.001).

The strongest staining for ERβ was found in the nuclei of the non-ciliated cells of the surface epithelium. The ciliated cells had a moderate to strong staining in the nuclei, but also some negative nuclei. Cytosolic staining was found in the surface epithelium. The cytosolic staining in the LB hens was more pronounced in the non-ciliated cells giving a stripy appearance of the surface epithelium compared to LSL hens (Figure 5a-c).

The tubular gland cells showed moderate to strong nuclear staining and moderate cytosolic staining. Strong nuclear staining was also found in the connective tissue cells. The endothelium of both capillaries and larger blood vessels showed strong nuclear staining. Erythrocytes were negative. Smooth muscle cells of muscularis and smooth muscle cells of blood vessels had strong nuclear staining. There was no other detectable difference in intensity or localization of ERβ between treatment, hybrids or interaction treatment and hybrid.

#### *Duodenum*

In duodenum strong staining for ERβ was found in the nuclei of surface epithelium, crypts of Lieberkühn and smooth muscle cells. There was a weak cytosolic staining for ERβ in crypts of Lieberkühn and surface epithelium, the staining gradually decreased towards the top of the villi. There was no detectable difference in intensity or localization of ERβ between treatment, hybrid or interaction treatment and hybrid.

### PMCA

The negative controls showed no staining of significance.

#### *Shell gland*

Strong staining for PMCA was found in the apical membrane of the tubular gland cells and weak staining was found apically in the surface epithelial cells. There were no detectable differences in intensity or localization of PMCA staining between treatments, hybrids or interaction treatment and hybrids.

#### *Duodenum*

Strong staining for PMCA was found in the basolateral membranes of the surface epithelial cells and the staining intensity was strongest at the top of the villi and gradually decreased towards the base. Weak basolateral membrane bound staining was found in the crypts of Lieberkühn cells. There were no detectable differences in intensity or localization of PMCA staining between treatments, hybrids or interaction treatment and hybrid.

## Discussion

Boosting laying birds with exogenous estrogen produces somewhat inconsistent results. Some are clearly related to dose and generally high doses reduce egg production with negative or no effect on shell quality, while plasma calcium increases [20,21]. By using two doses, 10 and 100 μg E2/kg bw, Saki *et al.,*[14] were able to show that the low dose improves shell quality without any adverse effects on egg weight or production, while the higher dose does not. It should be noted that the birds used by Saki *et al.,*[14] were Tegel pullets, a meat type breed. Since the main aim in our experiment was to study key factors involved in shell formation in the shell gland and duodenal calcium absorption, without disturbing normal production, the dose was calculated to be within the range of the lowest dose used by Saki *et al.,*[14]. To minimize the stress on the birds a subcutaneous pellet with a continuous release was chosen instead of daily injections.

In both hybrids laying percent, egg weight, plasma calcium, bw and oviduct length was unaffected by the estradiol treatment. Four shell quality parameters were improved in eggs from the brown LB hybrids; shell deformation, shell weight, shell thickness with and without shell membranes, while eggs from the white LSL birds showed an increase in shell thickness without shell membranes. Although significant, the increased shell thickness seen in our study was small (1% in LSL eggs and 2% in LB eggs). A dose of 10 μg E2/kg bw or a phytoestrogen supplement increases shell thickness by 9-11% [14,15], which is enough to reduce the percentage of cracked eggs in the experiment by Ni *et al.,*[15]. By fine-tuning of the dose to our hybrids the effect on shell thickness would most likely improve, but that requires a more applied approach which was not the intent of this study.

A direct action of estrogen on shell gland function in adult laying hens has not yet been demonstrated. Age related eggshell thinning has been associated with decreases in shell gland ERα [22] or possibly an imbalance between the two receptors. Both ERs were localized in the shell gland in the present experiment, with some differences between hybrids and how they reacted to the estrogen treatment. In both hybrids nuclear ERα was prominent in tubular gland cells, while the surface epithelium was negative except for slight granular cytosolic staining. ERβ, on the other hand, was intensely expressed in nuclei of surface epithelial cells and in tubular glands, both nuclear and cytosolic staining for ERβ was found of equal intensity in both hybrids. Especially cytosolic ERβ was more prominent in the shell gland surface epithelium of the brown hybrids, which also produced eggs with the largest improvement in shell quality after estrogen treatment. In addition, estrogen treatment decreased ERα in the white but not the brown hybrids. According to a recent review of physiological actions mediated via ERs in various tissues, the ratio of ERα and β within a cell may well determine its sensitivity to estrogens and consequently the biological response to the hormone [23]. An altered balance between the two receptors may therefore explain the different response after estrogen treatment seen in this study.

Estrogen is involved in duodenal calcium absorption and exogenous estrogen boosts calcium uptake in duodenal tissue according to Hansen *et al.*[24]. They used Compudose implants designed for cattle containing 24 mg of estradiol, likely providing a higher dose than our implants with 0.385 mg estradiol, which may explain why plasma calcium did not increase in our experiment. Estrogen is hypothesized to act in two different ways in the duodenum. One is by stimulating the conversion of vitamin D_3_ to 1,25 - dihydroxycholecalciferol, up-regulation of 1,25D_3_ receptors and the synthesis of calcium binding protein D28K in the duodenal mucosa [reviewed in [25]]. Estrogen is also suggested to act in a non-genomic manner, via extra-nuclear ERs and independent of vitamin D [26,27]. ERβ was the dominating ER detected in the duodenum and no differences appeared to exist between the two hybrids. The estradiol treatment did not alter localization or intensity of the staining and, both nuclear and cytosolic ERβ was found in the enterocytes.

Generally the brown hybrids had stronger staining for CA in duodenal brush border compared to the white hens. However, the only difference found after boosting with estrogen was decreased membrane-bound CA of the crypts in the brown LB hybrids. In normal female rats daily administration of estradiol increases duodenal CA activity [28] suggesting that this hormone may be involved in the regulation of duodenal CA activity to some extent. Preliminary data from both our own group and Nys and DeLaage [29] show a connection between lower duodenal CA activity and reduced eggshell quality. The role of CA in the mucosal defense mechanisms protecting enterocytes from the acidic chyme in the duodenal lumen has been extensively studied [30,31]. An interesting find is that perfusion of the lumen with a Ca^2+^ rich fluid decreases enterocyte intracellular pH, increases mucous gel thickness and HCO_3_^-^ secretion, mimicking the effect of luminal acidification. The effect is induced as Ca^2+^ is absorbed through the cells and stimulates the calcium sensing receptor (CaSR) at the basal membrane of the enterocytes [32], providing a possible explanation to how duodenal CA activity may affect shell quality.

An effect of estrogen boosting on CA activity was also seen in the shell gland where the proportion of CA positive capillaries increased in the white hybrids, while the brown hybrids were unaffected. In addition tubular gland CA activity also increased in the white birds but not the brown ones. The same hybrids given a feed supplement of a daidzein produce contradictory results, with capillary CA increasing in the brown LB hybrids but not the white [7]. These results suggest that estrogen has some regulatory influence on membrane bound CA activity in the shell gland, which is supported by the finding that estradiol up-regulates membrane bound CA in H9C2 cells [33]. Interestingly, only a specific dose-range of estradiol induces this effect in the H9C2 cells. Both our hybrids were exposed to the same dose but we have detected some differences in ER staining between the hybrids. It is known that both ERs exist in different isoforms and, at least the human ERα varieties (ER66 and ER46), show different binding affinity for a number of ER agonists, including the phytoestrogen daidzein [23,34]. Although more detailed experiments are needed in laying hens, these recent findings suggest that some contradictory results between hybrids may be explained.

In the shell gland the calcium transporter PMCA was localized in the apical membranes of tubular gland cells, which is in agreement with previous reports [7,35]. Staining intensity and localization of PMCA was unaffected by estrogen treatment in both hybrids. Calcium binding protein D28k is present in the tubular gland cells [36] and, with the addition of PMCA, these cells likely provide a possible route for the transport of Ca^2+^ needed for shell formation. Evidence is accumulating that the surface epithelium provides the bulk of the Ca^2+^ needed for shell formation [37,38]. The latest finding supporting this is the localization of transient receptor potential vanilloid channel type 6 (TRPV6) to the apical surface of these cells and that its mRNA increases in the shell gland during shell formation [39]. Since estrogen increases TRPV6 in human uterus [12] this is a very interesting candidate for future studies to elucidate the effects of estrogen on shell gland function.

PMCA was present in the basolateral membranes of the duodenal enterocytes. The localization fits with the hypothesis that calcium is extruded actively trough PMCA at the basolateral side of the enterocytes, after being absorbed passively through the apical cell membrane via TRPV6, localized in laying hen brush border membranes [40]. Although estrogen up-regulates PMCA in kidneys of laying hens and human uterus [12,13] no effects of estrogen could be detected in this study, which is in line with the unaltered plasma calcium concentration. The plasma calcium concentration did not differ between hybrids or after estrogen treatment, still the brown birds produced eggs with better shell quality than the white and presumably utilized more calcium in the process. Possibly, the white hybrids have a slightly different pattern in allocation of calcium between shell and bone, since bone strength was higher in the white hybrids compared to the brown LB birds.

## Conclusions

Boosting laying hens at the end of the laying period with a dose of estrogen, low enough not to affect plasma calcium, bw or egg production, improved shell thickness in both hybrids. In addition, shell weight and shell deformation improved in eggs from the brown hybrids. The more pronounced effect on the shell of eggs from the brown hybrids may be due to an increased sensitivity to estrogen, especially in surface epithelial cells of the shell gland, shown as an altered ratio between ERα and ERβ. A regulatory effect of estrogen on CA activity, but not PMCA, was seen in both duodenum and shell gland, and a possible connection to shell quality is discussed. Stronger femur was detected in LSL birds, suggesting that the hybrids differ in calcium allocation between shell and bone at the end of the laying period.

## Competing interests

The authors declare that they have no competing interests.

## Authors’ contributions

AW participated in the design of the study and was responsible for the practical arrangements, carried out the histological evaluation, collected all data, performed the statistical analysis and participated in drafting of the manuscript. LH was responsible for the design of the study and wrote the final draft of the manuscript in collaboration with YR. HW participated in the design of the study, assisted with the statistical analysis and was responsible for the animals. All authors have read and approved the final manuscript.
